# Excess costs of multiple sclerosis: a register-based study in Sweden

**DOI:** 10.1007/s10198-022-01547-6

**Published:** 2022-11-23

**Authors:** Chantelle Murley, Petter Tinghög, Fitsum Sebsibe Teni, Alejandra Machado, Kristina Alexanderson, Jan Hillert, Korinna Karampampa, Emilie Friberg

**Affiliations:** 1 Division of Insurance Medicine, Department of Clinical Neuroscience, Karolinska Institutet, 171 77 Stockholm, Sweden; 2 Department of Health Sciences, Swedish Red Cross University, 141 21 Huddinge, Sweden; 3 Division of Psychology, Department of Clinical Neuroscience, Karolinska Institutet, 171 77 Stockholm, Sweden; 4 Division of Neurology, Department of Clinical Neuroscience, Karolinska Institutet, 171 77 Stockholm, Sweden

**Keywords:** Multiple sclerosis, Cost of illness, Real world data, Disability pension, Sick leave, Primary healthcare, Healthcare costs, Productivity losses, Medical/health economics, Propensity score matching, I10, C55, D61, H51, H55, J10

## Abstract

**Background and objective:**

Population-based estimates of the socioeconomic burden of multiple sclerosis (MS) are limited, especially regarding primary healthcare. This study aimed to estimate the excess costs of people with MS that could be attributed to their MS, including primary healthcare.

**Methods:**

An observational study was conducted of the 2806 working-aged people with MS in Stockholm, Sweden and 28,060 propensity score matched references without MS. Register-based resource use was quantified for 2018. Annual healthcare costs (primary, specialised outpatient, and inpatient healthcare visits along with prescribed drugs) and productivity losses (operationalised by sickness absence and disability pension days) were quantified using bottom-up costing. The costs of people with MS were compared with those of the references using independent *t*-tests with bootstrapped 95% confidence intervals (CIs) to isolate the excess costs of MS from the mean difference.

**Results:**

The mean annual excess costs of MS for healthcare were €7381 (95% CI 6991–7816) per person with MS with disease-modifying therapies as the largest component (€4262, 95% CI 4026–4497). There was a mean annual excess cost for primary healthcare of €695 (95% CI 585–832) per person with MS, comprising 9.4% of the excess healthcare costs of MS. The mean annual excess costs of MS for productivity losses were €13,173 (95% CI 12,325–14,019) per person with MS, predominately from disability pension (79.3%).

**Conclusions:**

The socioeconomic burden of MS in Sweden from healthcare consumption and productivity losses was quantified, updating knowledge on the cost structure of the substantial excess costs of MS.

**Supplementary Information:**

The online version contains supplementary material available at 10.1007/s10198-022-01547-6.

## Introduction

Multiple sclerosis (MS) is the most common non-traumatic neurological disease among young adults [1]. Besides necessitating healthcare, MS may affect one’s work capacity. Accordingly, MS can pose an economic burden on patients, healthcare and social security budgets as well as a loss of production to society [2]. The all-age prevalence of MS in Sweden is relatively high at 189 per 100,000 [3, 4]. Therefore, accurate estimates of the socioeconomic burden of MS are essential to define the magnitude of the disease in monetary terms, justify interventions, and support decision makers in formulating policies and prioritising resources [2, 5].

The mean excess societal costs of MS in Sweden in 2012 from healthcare consumption and productivity losses were estimated in comparison with references to be 23,507 Euros (EUR) more per person with MS (2020 values) [6]. However, population-based cost estimates from Sweden, whether the all-cause costs among people with MS (PwMS) or the costs attributable to MS, have not yet included costs from primary healthcare use as such data is not available at a national level [6–9]. This has led to underestimations. Furthermore, studies on the primary healthcare utilisation among PwMS in Sweden have to date been of small groups, before the introduction of more efficacious disease-modifying therapies, and without reference groups [10–14]. Nonetheless, they suggest that PwMS have widespread primary healthcare use with a range of healthcare professionals [11]. PwMS are recommended to have a multidisciplinary care team [15]. Accordingly, primary healthcare has an important role in managing PwMS’ symptoms alongside neurology care [16]. Cost estimates need updating to reflect earlier diagnoses, increasing treatment options, and improved clinical course [17–19]. The excess costs of MS can be more accurately estimated with register data allowing for comparison with a reference group to isolate the additional cost per person due to MS and the underlying resource use may indicate unmet needs [20, 21].

We aimed to estimate the annual excess costs of MS, comparing the healthcare costs, including primary healthcare, and productivity losses among working-aged PwMS with those among population-based matched references.

## Methods and materials

### Study design and data sources

A one-year register-based cost-of-illness study was conducted. The resource utilisation, healthcare costs, and productivity losses of residents of Stockholm County, Sweden, with MS were compared with those of a population-based matched reference group without MS.

Individual-level data was linked from seven registers, using pseudonymised personal identity numbers, to build the study population and inform resource utilisation (Online Resource 1).

### Study population

**Fig. 1:**
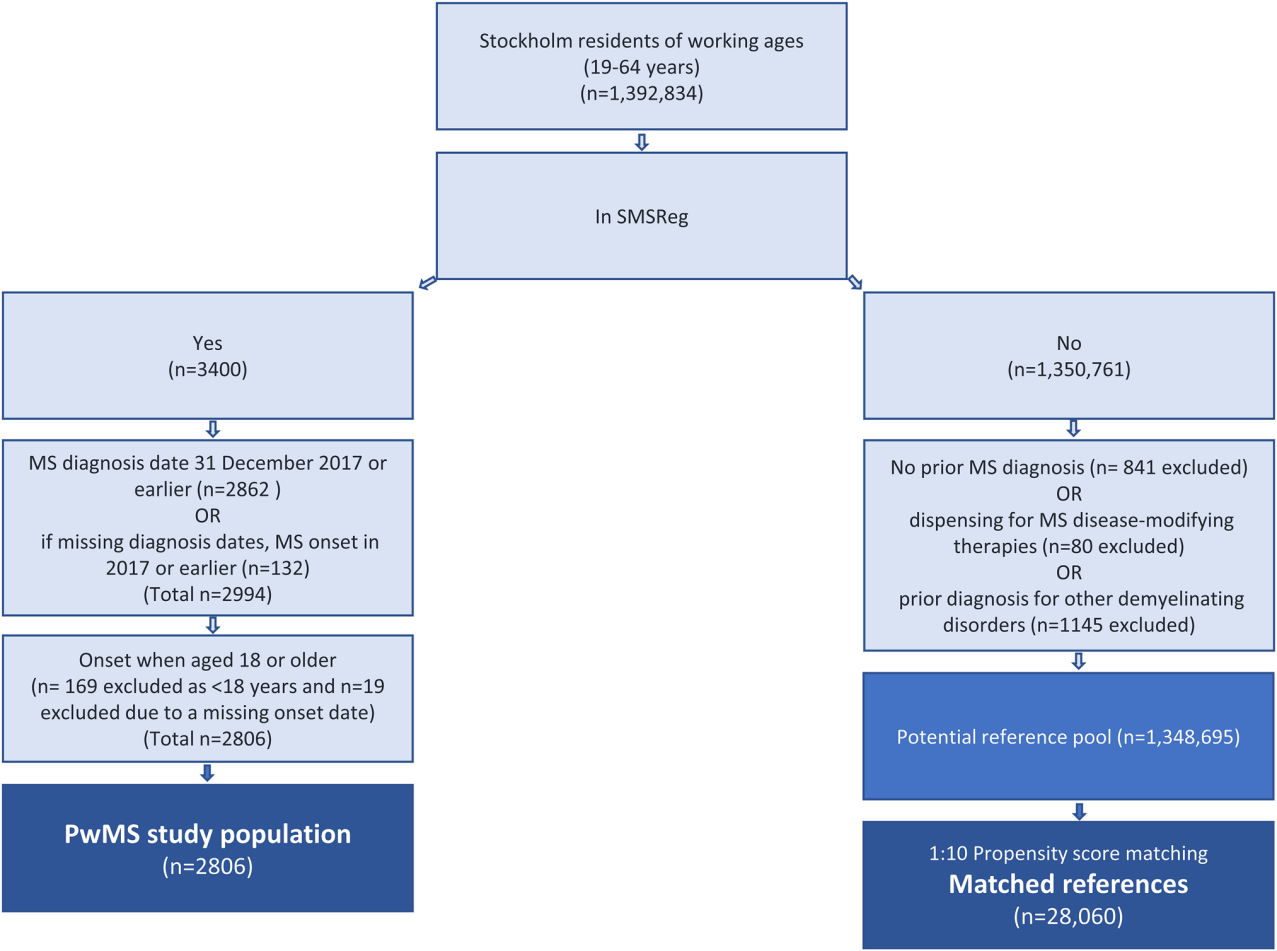
Flow chart of inclusion into the study population. Prior diagnosis of MS (ICD-10: G35 or ICD-8/9: 340) and other demyelinating diseases (ICD-10: G36, G37, H46, or ICD-9: 340, 377, 367, 323, 341) were identified in the NPR, VAL, or MiDAS [23–25]. MS disease-modifying therapies were identified in the SPDR (ATC codes: L01DB07, L01XC02, L01XC10, L03AB07, L03AB08, L03AB13, L03AX13, L04AA23, L04AA27, L04AA31, L04AA34, L04AA36, L04AA40, L04AC01, L04AX07, and N07XX09) [23, 25, 26]. *ATC* Anatomical Therapeutic Chemical Classification System, *ICD* International Classification of Diseases, *MiDAS* Micro Data for the Analysis of Social Insurance register, *MS* multiple sclerosis, *NPR* National Patient Register, *PwMS* People with multiple sclerosis, *SMSreg* Swedish Multiple Sclerosis Registry, *SPDR* Swedish Prescribed Drug Register, *VAL* Region Stockholm’s healthcare database. *Source*: Own elaboration

All individuals 19–64 years of age alive and living in Stockholm County on both 31 December 2017 (*n* = 1,392,834) were identified from the Longitudinal Integration Database for Health Insurance and Labour Market Studies (LISA) and cross-checked  for being alive and resident in Stockholm County in 2018 in LISA and the Cause of Death Register (*n* = 1,354,161) (Fig. 1).

The PwMS were identified from the Stockholm residents who were also in the Swedish MS Registry (SMSreg) (*n* = 3400), with an MS diagnosis recorded 31 December 2017 or prior (*n* = 2862). Of the 179 in the SMSreg but with a missing MS diagnosis date, 132 with onset dates before the end of 2017 were included. Lastly, individuals with MS onset when < 18 years were excluded as their clinical course may differ from adult-onset MS (*n* = 188 excluded) [22]. In total, 2806 PwMS were included.

A population-based matched reference group was identified from the Stockholm residents, who have never been diagnosed with MS (not included in the SMSreg, *n* = 1,350,761; Fig. 1). Individuals were then excluded from being potential references if having diagnosis codes (main or secondary) for MS or other demyelinating diseases [23–25] in the National Patient Register (NPR), Region Stockholm’s healthcare database (VAL), or the Micro Data for the Analysis of Social Insurance register (MiDAS), or MS disease-modifying therapies [23, 25, 26] dispensed according to the Swedish Prescribed Drug Register (SPDR) up until 31 December 2018 (*n* = 2066 excluded). From this pool of potential references (*n* = 1,348,695), propensity-score matching selected ten reference individuals for each individual with MS (*n* = 28,060) [27–30].

### Resource use and costs

A prevalence-based costing approach was used to estimate the costs incurred within the year attributable to MS from a societal perspective [31, 32]. Total costs of all-cause resource use for each cost category were estimated through a bottom-up approach by multiplying each individual’s total quantity of resource use in 2018 with the corresponding unit costs summarised in Table 1 [32]. A reference year of 2020 was used whenever possible for the unit costs [32, 33]. Costs are presented in EUR (1 EUR = 10.4848 Swedish krona (SEK)) [34].

**Table 1:** Unit costs^1^ for the register-informed resource use for the cost calculations for healthcare and productivity losses. *Source*: Own elaboration with sources referenced

Cost category	Value in 2020 SEK^1^	Value in 2020 EUR^2^	Source
(1) Healthcare costs			
(1a) Healthcare visits			
Average inpatient and specialised outpatient healthcare cost per 1.0 DRG	56,356	5375	The unit cost for 1.0 retrospective nationwide DRG weight for somatic care in 2018 was from the Swedish Association of Local Authorities and Regions and inflated to 2020 values [37]
Copayment per day of an inpatient stay	100	10	An out-of-pocket cost of 100 SEK per day was assumed for inpatient healthcare, as this was the base case for Region Stockholm [36]. Patient out-of-pocket costs for inpatient stays were capped at 365 days
Copayment for a specialised outpatient healthcare visit	350	33	An out-of-pocket cost of 350 SEK per visit was applied as the copayment for a visit in Region Stockholm to a physician in specialised outpatient settings [35]. The maximum copayment amount for specialised outpatient healthcare was set to 1150 SEK (109 EUR) which was the ceiling price for Region Stockholm within 12 months for specialised outpatient and primary healthcare [35]
Average cost per primary healthcare visit (at a clinic with a physician)	1879	179	The unit cost per contact with a physician at a primary healthcare clinic was inflated to 2020 values from an average 2019 unit cost and the unit cost ratio was sourced from Swedish Association of Local Authorities and Regions [40]. A home or off-site visit (hereafter referred to as simply home visits) had a unit cost corresponding to 2 clinic visits and a distance contact (including a telephone prescription) [42] was 1/3 of a clinic visit. The missed appointments were included within clinic visits to be costed due to the opportunity costs they represent (331 missed appointments among the PwMS and 2360 among the references). Visits with other healthcare professionals than physicians (e.g., nurses) corresponded to 40% of the relevant type of visit with a physician. The cost included all professionals listed for the contact (maximum of 5) with the costs attributed to the first registered professional
Copayment for a primary healthcare visit	100 or 200	10 or 19	Patient copayments for the contact were based on the first registered professional. A cost of 200 SEK was applied to physicians, physiotherapists, dieticians, occupational therapists, chiropractors, or naprapaths. All other healthcare professionals had a cost of 100 SEK per contact. These unit costs were sourced from the listed patient fees for Region Stockholm [35]. The maximum copayment amount for primary healthcare contacts was set to 1150 SEK (109 EUR) per year which was the ceiling price for Region Stockholm within 12 months for specialised outpatient and primary healthcare [35]
(1b) Drugs^3^			
Monthly cost for rituximab	1348	129	Rituximab is used off-label for MS in Sweden [47]. Rituximab is sold as 500 mg units with a price per unit of 9704 SEK [48]. A treatment frequency of rituximab of every 7.2 months was applied based on a previous observational study [49]. The dose per treatment was assumed to be 500 mg based on the SMSreg data as the most frequent dosing in 2018 (93.96%) and the recommendation from the Swedish MS Association [47]. The cost per month of rituximab (1348 SEK) was assumed to be the unit cost divided by the frequency of treatment. Initiation of rituximab is always 1000 mg [47], hence, an extra cost of 9704 SEK was applied (i.e., an additional 500 mg unit) if the year of treatment initiation was 2018 (*n* = 159)
Monthly cost for natalizumab	15,839	1511	Treatment frequency with natalizumab is every 4th week (i.e., once per month), with a dose of 1 unit (300 mg) [50]. The cost per month of natalizumab was assumed to be the price of a dose (300 mg) of natalizumab (15,839 SEK) [50]
Monthly cost for alemtuzumab	18,368	1752	Alemtuzumab is sold as 12 mg units at an assumed price of 73,473 SEK [51]. A unit of 12 mg equates to 1 dose [52]. Starting treatment involves 5 doses (60 mg) with maintenance of 3 doses (36 mg) every 12 months thereafter, for up to a maximum of 3 additional cycles to the starting cycle [52, 53]. A full cycle of use was assumed from a start date in the SMSreg. Accordingly, a monthly cost of treatment with alemtuzumab of 18,368 SEK was applied, with an add on start cost of 146,947 SEK (corresponding to the cost of the 2 extra doses for initiation) if the year of treatment initiation was during 2018 (*n* = 1)
Monthly cost for ofatumumab	3616	345	Ofatumumab was used off-label for MS in 2018. Ofatumumab was sold as 100 mg units at an assumed price of 7233 SEK [54] before deregistration for this dosage and administration form in 2019 [55]. A 300 mg dose every 6 months was assumed based on the SMSreg data. A monthly cost of 3616 SEK was applied for ofatumumab from averaging 50 mg per month on treatment
Monthly cost for ocrelizumab	18,182	1734	Ocrelizumab is sold as 300 mg units at an assumed price of 54,546 SEK [56]. 300 mg equates to 1 dose [57]. Treatment initiation involves 1 dose in week 1 and a further dose in week 3 with a maintenance protocol of 2 doses (600 mg) administered every 6 months from treatment initiation (week 1) [57, 58]. A monthly cost of 18,182 SEK was applied for ocrelizumab from averaging 100 mg per month on treatment
(2) Productivity losses^4^			
Monthly salary including the employer contributions	47,443	4525	The average value of a month of production was inferred from the mean monthly salary including employer contributions. The mean monthly salary for all sectors in 2020 was retrieved from Statistics Sweden [45]. The employer social security contributions for 2020 (31.42%), available from the Swedish Tax Authority, were then added to the mean monthly salary to determine the average value of a month of lost production [46]

*DRG* diagnosis-related group, *EUR* Euro, *PwMS* People with multiple sclerosis, *SEK* Swedish krona, *SMSreg* Swedish Multiple Sclerosis Registry

^1^Unit costs were for 2020. Where this was not possible, the most recent unit cost was inflated to 2020 SEK values with the annual Harmonised Indices of Consumer Prices for healthcare [59]

^2^Conversion to EUR to facilitate international comparisons with conversion via the 2020 annual exchange rate of 10.4848 [34]

^3^Drug costs also included the costs for prescribed drugs dispensed in community pharmacies. The costs of these drugs were collected directly from the register

^4^Productivity losses are presented as sickness absence costs and disability pension costs, based on the social insurance used to infer the months of hypothetically lost production

#### Healthcare costs

Healthcare costs included the costs of healthcare visits (inpatient, specialised outpatient, and primary healthcare) and drugs (prescribed drugs dispensed in pharmacies and MS disease-modifying therapies administered within healthcare).

The costs for both inpatient (with a discharge date in 2018) and specialised outpatient visits, respectively, were derived from the diagnosis-related group (DRG) code for the visit in the NPR along with the patient copayment [35, 36]. Nationwide retrospective DRG weights were used to translate the DRG code into a cost [37, 38]. If the DRG was not in the retrospective weight list for 2018 (*n* = 355 inpatient and *n* = 19,248 specialised outpatient visits), the prospective nationwide DRG weight for 2018 from the National Board of Health and Welfare was used instead [39].

Primary healthcare contacts were sourced from the VAL and classified according to the type of contact and healthcare professional. The contacts were then costed relative to the average cost for a clinic visit with a physician (Online Resource 2) and the relevant patient copayment [35, 40–42]. Primary healthcare was further analysed. The type of healthcare visit was presented as clinic (116,032 visits including 2691 missed appointments), home, and distance contact. Type of healthcare professional referred to the first registered professional, with 91.9% of contacts with 1 professional. Two categorisations were used:

1. Physicians, nurses, or other healthcare professionals; and
2. Physicians, nurses, physiotherapists, occupational therapists, nurse assistants, or other healthcare professionals.

Drug costs comprised the costs for prescribed drugs dispensed in community pharmacies and other MS disease-modifying therapies administered within healthcare. Drug costs related to the quantity of the specific substance dispensed, with the patient copayment [43] and the portion of the costs publicly financed all obtained directly from the SPDR*.* In addition, further MS disease-modifying therapies administered within healthcare and not available in the SPDR were included. Start and end dates of treatment from the SMSreg were used to estimate the months on treatment. Average unit costs per month were applied based on recommended dosing. Drug costs were also presented as MS disease-modifying therapy costs (sourced from either the SPDR or SMSreg) and drug costs excluding disease-modifying therapies.

#### Productivity losses

The costs of productivity losses were estimated from the net days of sickness absence and disability pension from MiDAS, representing days with lost production to society due to morbidity [31]. The human capital approach was used to estimate productivity losses, as per the Swedish guidelines for economic evaluations [31, 44]. Productivity losses were derived by multiplying the net months of sickness absence and disability pension (months with lost production), respectively, by the sum of the national average monthly gross salary and the employers’ social security contribution [45, 46]. Full employment of the study population was assumed.

#### Total societal costs

Annual total societal costs per person were the sum of healthcare costs and productivity losses.

### Statistical analyses

Propensity score matching was used in this observational study to mimic randomisation by constructing a reference group with a similar covariate distribution to the PwMS to reduce bias in the estimation of the costs of MS [27–29]. This matching strategy was deemed advantageous as it provided intuitive estimates of the cost of MS by design rather than conditioned on a subgroup of PwMS in subsequent analyses, and it allowed for simpler statistical analyses with reduced variance in the estimates [27, 29]. Furthermore, it can be problematic to directly adjust for comorbidities in register-based MS research due to difficulty in disentangling whether another diagnosis is unrelated or connected as a symptom or consequence of MS [21, 60]. Adjustment for diagnoses related to MS would be an overadjustment and lead to underestimations of the excess costs of MS.

The propensity score was estimated with a logistic regression model with MS status (MS vs. no MS) as the outcome and the following baseline factors, with information from 31 December 2017, as independent variables: sex (women, men), age (years), born in Sweden (yes, no), degree of urbanisation of their municipality of residence (binary variables for each, cities: densely populated areas; towns and suburbs: intermediate density areas; and rural areas: thinly populated areas), educational level dichotomised as university education (yes, no), and family composition classified as child living at home (yes, no) and cohabiting or married (yes, no). The predicted probabilities of MS status (propensity scores) were then used to create a matched sample of references. We used a calliper width of 0.25 and the greedy nearest neighbour matching method without replacement [27–29]. To determine covariate balance between the PwMS and matched references, we calculated standardised mean differences for each included baseline factor before matching among all potential references (*n* = 1,348,695) in comparison with the PwMS and then reassessed the standardised mean differences for the 28,060 selected references. The threshold to indicate imbalance was set at > 0.1 [61, 62]. Common support was assessed by comparing the propensity score distributions of the PwMS and references.

After the propensity score matching, descriptive statistics of the matched study population were conducted. All individuals were included in the statistical analyses to enable inference on the entire study population, including those with zero costs. Costs were reported as annual means per person with 95% confidence intervals (CIs). The estimated population mean is widely thought as the cost statistic of most interest to policy makers and easily interpretable [63]. Median values and interquartile ranges (IQR) are also reported given the characteristic right-skewed cost data with a mass of zero observations [63]. Among all, 16.6% of individuals accrued 0 healthcare costs (PwMS = 2.1%, references = 18.0%) and 81.6% had 0 productivity losses (PwMS = 51.1%, references = 84.6%) within 2018. The relative difference in mean per person costs was calculated as a ratio by dividing the costs among PwMS with those among references. The proportions of individuals with resource use underlying each cost category were presented (yes, no). Differences in resource use between the PwMS and the matched references were tested with chi-square tests.

Excess costs were the costs of MS, isolated by comparing the all-cause costs among PwMS with those of the matched references [21]. Accordingly, excess costs were the mean cost differences between the PwMS and the references for each cost category that we calculated with direct comparisons from two-tailed Student’s *t*-tests. These excess cost estimates without adjustment provide a mean value across all individuals, irrespective of age, sex, or other characteristics [64]. The relatively large study population and study design with matched references enabled relatively simple analyses [63]. Furthermore, further adjustment with regression modelling was not considered necessary to remove residual confounding after the propensity score matching, as none of the observed covariates indicated imbalance (standardised mean differences > 0.1), suggesting a well matched reference group [62]. Lastly, bootstrapped bias-corrected percentile CIs for the excess cost estimates were computed from 2000 iterations to indicate the plausible range of values for the population parameter [65].

Data management (except for costing in STATA v15) and statistical analyses were performed using SAS v.9.4. The Regional Ethical Review Board of Stockholm, Sweden approved the project.

## Results

### Study population

The mean age of the PwMS was 45.3 years (95% CI 44.9–45.7). Through propensity score matching, 28,060 residents in Stockholm without MS were matched to the 2806 PwMS. All baseline factors included in the propensity score had standardised mean differences < 0.1 after matching (Table 2) and the distributions of the propensity scores overlapped (Online Resource 3).

**Table 2:** Standardised mean differences between the people with multiple sclerosis (PwMS) and references without multiple sclerosis before and after matching. *Source*: Own elaboration

	Variable^a^	PwMS (*n* = 2806)		Reference pool (*n* = 1,348,695)			Matched references (*n* = 28,060)		
		*n*	%	*n*	%	Standardised mean difference	*n*	%	Standardised mean difference
Propensity score	Mean (SD)	0.003 (0.002)		0.002 (0.001)		0.602	0.003 (0.002)		0.000
Sex						0.44			0.000
	Women	1969	70.20	662,323	49.10		19,690	70.17	
	Men	837	29.83	686,372	50.89		8370	29.83	
Born in Sweden^b^						− 0.244			− 0.001
	No	554	19.74	407,592	30.22		5550	19.78	
	Yes	2252	80.30	941,103	69.80		22,510	80.22	
University education^c^						− 0.048			− 0.001
	No	1365	48.65	688,329	51.04		13,670	48.72	
	Yes	1441	51.20	660,366	48.96		14,390	51.28	
Married or cohabiting						− 0.124			0.001
	No	1339	47.72	727,177	53.92		13,370	47.65	
	Yes	1467	52.30	621,518	46.10		14,690	52.35	
Living with a child (< 18 years)						− 0.019			0.000
	No	1784	63.58	869,474	64.47		17,840	63.58	
	Yes	1022	36.40	479,221	35.50		10,220	36.42	
Age^d^						0.409			− 0.001
	19–24 years	53	1.89	154,612	11.46		530	1.89	
	25–34 years	485	17.28	338,747	25.12		4850	17.28	
	35–44 years	753	26.84	321,518	23.84		7520	26.8	
	45–54 years	868	30.93	304,327	22.56		8680	30.93	
	55–64 years	647	23.06	229,491	17.02		6480	23.09	
Degree of urbanisation of municipality of residence									
	Cities	1943	69.24	948,841	70.35	0.024	19,455	69.33	0.002
	Towns and suburbs	730	26.02	343,640	25.48	− 0.012	7305	26.03	0.000
	Rural areas	133	4.74	56,214	4.17	− 0.028	1300	4.63	− 0.005

*PwMS* People with multiple sclerosis, *SD* standard deviation

^a^Variables measured on the 31 December 2017

^b^Missing values were categorised as no (*n* =  < 10, 0.00% in the included study population)

^c^Missing values were categorised as no (*n* = 386, 1.25% in the included study population)

^d^Included as years within the propensity score

### Costs of PwMS

The mean annual costs for healthcare resource consumption and productivity losses of PwMS and matched references are visualised in Online Resource 4 and detailed in Online Resource 5. Productivity losses were the largest cost for both the PwMS (16,922 EUR per person, 95% CI 16,101–17,742) and references (3748 EUR, 95% CI 3608–3888), with an average 4.5-fold higher productivity loss among PwMS. The corresponding mean annual healthcare costs were 9595 EUR (95% CI 9189–10,000) per person with MS and 2214 EUR (95% CI 2146–2282) for the references, equating to 4.3-fold higher healthcare costs among PwMS.

### Excess costs of MS and differences in resource utilisation

**Fig. 2:**
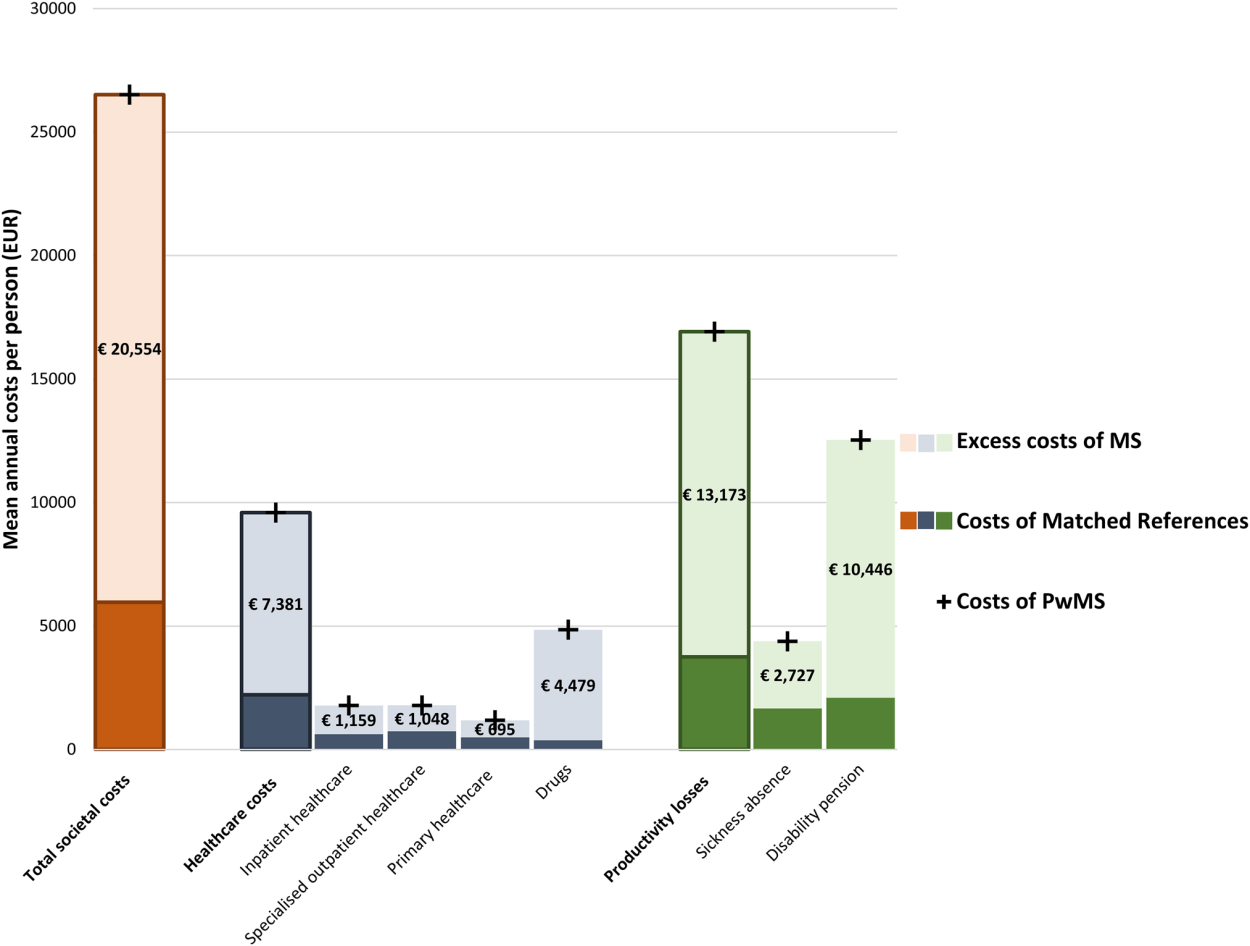
Annual mean excess costs of MS per person for healthcare consumption and productivity losses, as well as the sum of all-cause costs among PwMS and their propensity score matched references without MS. *Notes*: Total societal costs are in orange, healthcare costs and component costs are displayed in blue, and the productivity losses and component costs are displayed in green. The paler of the respective colour represents the excess costs of MS. The costs stated refer to these excess costs of MS. Each entire column represents the sum of all-cause costs among PwMS and the darker portion of the column the costs among the matched references without MS. The annual exchange rate for 2020 from SEK to EUR from Eurostat was 10.4848 [34]. Prior to currency conversion, if required, the unit costs were inflated to 2020 Swedish prices using the annual Harmonised Indices of Consumer Prices for healthcare available from Eurostat [59]. *EUR* Euros, *MS* multiple sclerosis, *PwMS* People with multiple sclerosis, *SEK* Swedish krona. *Source*: Own elaboration

The excess costs of MS with bootstrapped 95% CIs are visualised in Fig. 2 and summarised in Table 3. There was a mean annual excess productivity loss of MS of 13,173 EUR (95% CI 12,352–14,019) per person with MS. Disability pension costs comprised 79.3% of this excess. More PwMS had productivity losses (48.9%) than references (15.3%; *p* value < 0.001) (Online Resource 6).

**Table 3:** Summary of the excess costs of MS per person^a^ from mean differences in the costs among PwMS (*n* = 2806) and propensity-score matched references (*n* = 28,060) with bootstrapped 95% CIs. *Source*: Own elaboration

Cost category	Excess per person costs of MS (EUR)	(95% CI)	*p* value	Percentage the cost category contributes
Total societal costs	20,554	(19,552 to 21,549)	< 0.001	
Healthcare costs	7381	(6991 to 7816)	< 0.001	100.00
Inpatient healthcare costs	1159	(928 to 1394)	< 0.001	15.70
Specialised outpatient healthcare costs	1048	(959 to 1133)	< 0.001	14.20
Primary healthcare costs	695	(585 to 832)	< 0.001	9.42
Drug costs	4479	(4224 to 4769)	< 0.001	60.68
Productivity losses	13,173	(12,325 to 14,019)	< 0.001	100.00
Sickness absence costs	2727	(2278 to 3186)	< 0.0001	20.70
Disability pension costs	10,446	(9636 to 11,206)	< 0.0001	79.30

*CI* confidence interval, *EUR* Euros, *MS* multiple sclerosis, *PwMS* People with multiple sclerosis, *SEK* Swedish krona

^a^The annual exchange rate for 2020 from SEK to EUR from Eurostat was 10.4848 [34]. Prior to currency conversion, the unit costs were inflated to 2020 Swedish prices using the annual Harmonised Indices of Consumer Prices for healthcare available from Eurostat [59]

Differences in the proportions of users of healthcare resources and excess costs of MS for healthcare were observed. Among PwMS, specialised outpatient healthcare settings had the most users (79.6% PwMS vs. 48.9% references, *p* value < 0.001). References had most users in primary healthcare settings but at a lower proportion than among the PwMS (65.5% references vs. 75.9% PwMS, *p* value < 0.001). A mean annual excess cost of MS for healthcare of 7381 EUR (95% CI 6991–7816) was discerned per person with MS. While PwMS had a higher percentage of their total annual healthcare costs publicly funded (96.8% vs. 90.4%), there was a mean annual excess cost for visit fees and drugs paid out-of-pocket of 91 EUR (95% CI 73–107) per person with MS. There was a mean annual excess drug cost of 4479 EUR per person with MS (95% CI 4224–4769). Disease-modifying therapies contributed 95.2% of these excess costs.

### Primary healthcare utilisation and costs

**Fig. 3:**
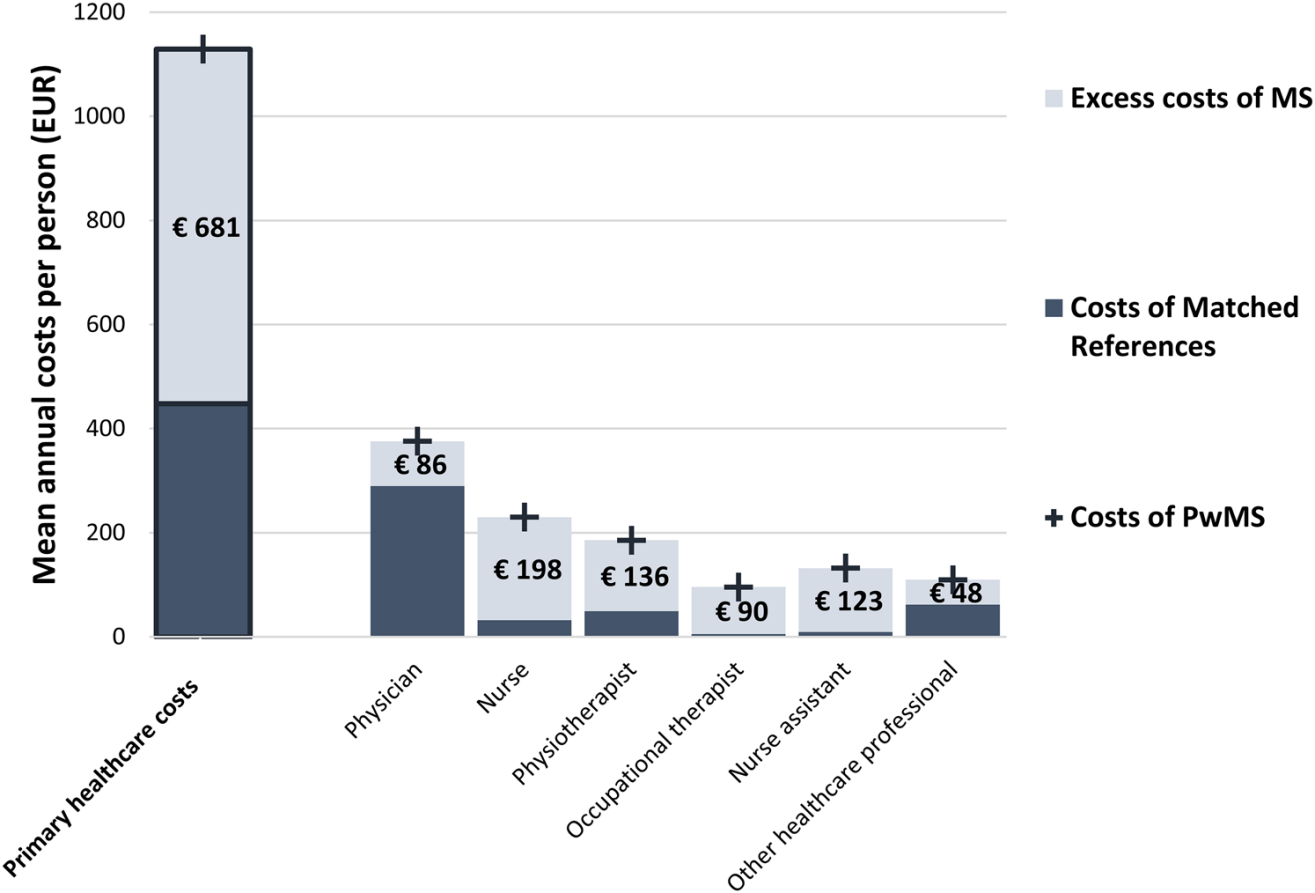
Annual mean excess costs of MS per person for primary healthcare by healthcare professional. *Notes*: The paler colour represents the excess costs of MS. The costs stated refer to these excess costs of MS. Each entire column represents the sum of all-cause costs among PwMS and the darker portion those among the matched references without MS. The first registered healthcare professional per contact is used. Primary healthcare costs in this figure are presented excluding the patient out-of-pocket copayment in the form of visit fees. The mean annual cost per person for copayments for primary healthcare were estimated to be 52 EUR (95% CI 51–54) for PwMS and 38 EUR 95% CI 38–39) for the references. The annual exchange rate for 2020 from SEK to EUR from Eurostat was 10.4848 [34]. Prior to currency conversion, the unit costs were inflated to 2020 Swedish prices using the annual Harmonised Indices of Consumer Prices for healthcare available from Eurostat [59]. *CI* confidence interval, *EUR* Euros, *MS* multiple sclerosis, *PwMS* People with multiple sclerosis, *SEK* Swedish krona. *Source*: Own elaboration

The excess primary healthcare costs of MS are presented by healthcare professional in Fig. 3 and tabulated in Online Resource 7. Primary healthcare costs were 2.4-times higher per person with MS, with a mean annual cost for primary healthcare of PwMS of 1181 EUR (95% CI 1060–1302) per person and 486 EUR (95% CI 475–498) for the references. This resulted in a mean annual excess cost of 695 EUR (95% CI 585–832) per person with MS. Primary healthcare constituted 9.4% of the excess healthcare costs of MS. A higher proportion of PwMS had primary healthcare visits at the clinic (74.3% vs. 65.3%, *p* value < 0.001) or home (13.8% vs. 1.2%, *p* value < 0.001) compared with references, but similar proportions had distance contacts (3.78% vs. 3.74%, *p* value 0.917). Excess costs of MS for clinic visits (237 EUR, 95% CI 197–274), home visits (444 EUR, 95% CI 342–560) were observed but no excess costs of MS for distance contacts (*p* value 0.830). Physicians were the most contacted healthcare professional among PwMS (66.5%) and references (58.5%, *p* value < 0.001). Higher proportions of PwMS than references had contact with nurses, physiotherapists, occupational therapists, and nurse assistants (*p* values < 0.001). Excess costs of MS were observed for all investigated healthcare professionals. Having MS was associated with a mean annual excess cost per person of 86 EUR (95% CI 66–107) for primary healthcare contacts with physicians, 198 EUR (95% CI 146–251) with nurses, and 397 EUR (95% CI 325–493) with other healthcare professionals.

## Discussion

This population-based register study quantifies the socioeconomic burden of MS in Sweden as the excess costs of MS calculated in comparison with the costs of propensity-score matched references. The mean annual excess costs were 7381 EUR for healthcare and 13,173 EUR for production losses per working-aged person with MS. In relative terms, this equated to 4.3-fold higher healthcare costs and 4.5-fold higher productivity losses per person with MS. PwMS had a mean annual excess cost of 695 EUR per person for primary healthcare, mostly from contacts with healthcare professionals other than physicians. The excess costs of MS outside the health system were substantial, especially due to lost productivity when disability pensioned.

Our findings update previous estimates from Sweden. Gyllensten et al. found that the mean excess costs of MS for working-aged PwMS were lower in 2012 at 8807 EUR for healthcare costs and 14,700 EUR for productivity losses, than in 2006 (2020 values) [6]. Our estimates are slightly lower again, notwithstanding inclusion of primary healthcare. The excess costs of MS were driven by disease-modifying therapies and permanent reductions of work capacity (disability pension costs), consistent with previous research [6, 66]. In addition to earlier diagnoses [19], new treatments have entered the market [17] changing healthcare utilisation, e.g., moving from inpatient to outpatient healthcare, as well as reducing MS disability. Accordingly, offsets of these treatment costs from savings of other healthcare resources [67] and maintained work capacity [6] have been suggested. Economic modelling also supports the notion that early initiation of treatment is cost saving to society [68]. This finding was robust across models, including a Sweden-specific model, and more costs were saved from averting MS the longer the time horizon [68]. Our relatively low excess healthcare costs of MS could also be influenced by rituximab being widely used off-label [17]. We observed 43.3% with rituximab infusions within 2018. The high use of rituximab, especially among PwMS with high inflammatory activity or relapses [47], influences our cost estimates given the lower unit costs compared with alternative treatments available.

Our study is unique in quantifying the excess costs of MS for primary healthcare from real-world resource consumption. These costs comprised 9% of the excess healthcare costs of MS, updating knowledge on the cost structure of MS. We observed widespread primary healthcare utilisation by PwMS and excess primary healthcare costs of MS were largely for professionals other than physicians, consistent with the recommendations for multidisciplinary care to manage MS and the wide range of symptoms [15]. Therefore, indicating not just more but a different pattern of utilisation by PwMS. In a previous study of 121 PwMS in Stockholm County, nurses, physicians, physiotherapists, and occupational therapists were the most sought after primary healthcare professionals over 10 years of follow-up [14]. With a single observation year, the same professionals each represented > 5% of all primary healthcare contacts among the PwMS, with the addition of nurse assistants. In any country, healthcare for PwMS is provided by both primary care and specialised units, in particular neurology departments or even MS-specific centres. In Sweden, there are roughly 60 neurology centres providing specialised MS healthcare. This study shows that in Stockholm, primary care for PwMS includes rehabilitative services by occupational therapists and physiotherapists and care provided by nurses with smaller additional costs for primary healthcare physicians. Hence suggesting that neurologists provide not only specialised MS care but also service more general medical needs of PwMS. It is possible that this pattern of healthcare use is more prominent in Stockholm, owing to the high concentration and proximity to neurologists, compared with smaller towns and rural areas.

Excess costs of MS were observed for all healthcare settings. The excess costs of MS for primary healthcare were of a smaller magnitude than for the higher cost settings of specialised out- and inpatient healthcare. PwMS often have parallel use of healthcare services in different departments and types of healthcare [11]. Future research should investigate the department, procedures, and diagnoses contributing to the excess healthcare costs of MS and underlying changes in productivity in the era of modern therapy [14].

The socioeconomic burden of MS reaches beyond the healthcare system [66]. Productivity losses increasingly outweigh the healthcare costs with worsening MS, as MS particularly affects young adults with the potential for many more years of paid work [7, 66]. Our excess productivity losses of MS were larger than previous estimates from newly-diagnosed PwMS and predominately from permanent work incapacity [9]. Whereas the mean annual excess sickness absence costs around MS diagnosis were larger than observed in our prevalent cohort [9]. The excess productivity losses owing to sickness absence and disability pension reflect challenges that PwMS face to maintain work capacity and remain in or return to work. These excess costs of MS can indicate unmet needs among this population regarding morbidity as well as work adaptions. Interventions, including early initiation of high-efficacy disease-modifying therapies, to promote the maintenance of work capacity of PwMS are required to address this unmet need and high costs.

The major strengths of this study were the population-based design and the use of high-quality register microdata [69–72]. The excess costs of MS mirrored real-world resource utilisation and were not dependent on recall. We had a relatively large study population compared with previous Swedish studies of primary healthcare use among PwMS [11, 12, 73]. Further, a reference group from the same source population was included to calculate the excess costs of MS. Excess costs are well suited for isolating the costs of MS [21]. Comorbidities are common among PwMS and may also influence wider resource use, with complexity as to the causal relationship with MS [60, 74]. It is not always apparent which diagnosis the resources should be attributed to [21, 60]. Hence estimates of excess costs through comparisons with a reference group have a lower risk of classification errors owing to whether the wider register-based resource use was due to MS, or not, than costs informed only by resource use coded with an MS diagnosis [6]. Accordingly, we were able to estimate the excess costs directly and indirectly related to MS, to accurately measure the wider costs of MS. Propensity-score methods are increasingly used in MS and cost-of-illness studies with observational real-world data to reduce confounding and account for individual-level factors in expected resource use [21, 28, 61, 75]. By achieving covariate balance after matching, we avoided the need for double adjustment through modelling [62]. However, there is potential for residual confounding from other factors, whether unmeasured or unknown [29].

Our findings should be interpreted considering limitations. The PwMS were sourced from the SMSreg, which has high coverage [76]. However, 841 individuals were removed as potential references for having an MS code in another register. These individuals may have differed to those in the SMSreg, e.g., individuals with MS codes for primary healthcare or disability pension may have been older, have progressive MS, and not be treated with disease-modifying therapies. Rituximab was an exclusion for potential references because of the high frequency of use for MS but possibly excluded individuals not having MS [17, 48]. Optic neuritis (H46), one of the other demyelinating diseases, was unable to be identified from the NPR.

The main limitation is the lack of register data for both PwMS and references regarding other relevant cost categories, including community services, adaptations, informal care, reduced productivity at work, as well as productivity losses from sickness absence spells ≤ 14 days. The true excess costs of MS are likely to be higher with these costs included.

For the included costs, there are also important considerations. Healthcare costs were largely based on aggregate average unit costs as proxies for the actual costs [38]. These should cover the standard treatment burden and foster comparison with other studies, but may underestimate costs in situations requiring higher than average resources. To avoid temporal biases from the rearrangements of healthcare during the COVID-19 pandemic, earlier unit costs were used for healthcare visits and inflated to 2020 values. If the routines implemented continue, these costs are accordingly underestimated. The administration costs for disease-modifying therapies are presumed to be included within the specialised outpatient healthcare costs, although visits without involvement of a physician were not recorded in the data and consequently the full treatment costs are underestimated. There is also a slight overestimation of patient copayments from including separate caps for specialised outpatient and primary healthcare visits. Lastly, only primary healthcare financed by Region Stockholm was captured in the data, leading to underestimations of care sought elsewhere.

The generalisability of the excess cost estimates and resource use may be limited to settings with similar universal coverage for social security and healthcare and labour market/job security regulations. The observed patterns of resource utilisation and resulting costs reflect the healthcare organisation, medical traditions, and access of the working-aged individuals with adult-onset MS in a largely urban area. Sweden has a comparatively low average number of hospital beds per capita as well as physician consultations [77], the latter suggested to be related to the central role of nurses [78]. The regional financing and provision of healthcare in Sweden may also influence the service availability and utilisation patterns [79]. There are also likely differences in the magnitude of costs and distribution of cost components among the PwMS, for example, by disability level [7, 66] or among those with late onset MS [80, 81]. The latter being much less studied. The excess costs of MS presented are the additional cost on top of regular expenditure per individual. The excess costs of MS are considerable with approximately 20,000 working aged PwMS throughout Sweden [82].

## Conclusions

In this population-based study with real-world data, costs for healthcare consumption and lost production were compared between a prevalent cohort of working-aged people with MS and propensity-score matched references to estimate the magnitude of the socioeconomic burden of MS. PwMS had substantially higher costs from healthcare use, including primary healthcare, and production losses than references. Primary healthcare contributed a tenth of the excess healthcare costs of MS, largely from contacts with professionals other than physicians, updating knowledge on the cost structure of MS. The excess costs of MS from lost production in this prevalent MS cohort were larger in magnitude than healthcare consumption. The substantial excess costs of MS indicate potential unmet needs in the MS population. Interventions to reduce morbidity and support PwMS to stay or return to work are vital.

### Supplementary Information

Below is the link to the electronic supplementary material.

Supplementary file1 (PDF 350 kb)

## Data Availability

The sensitive microdata used in this study is administered by the Division of Insurance Medicine, Karolinska Institutet, and cannot be shared publicly in accordance with the General Data Protection Regulation, the Swedish Data Protection Act, the Swedish Ethical Review Act, and the Swedish Public Access to Information and Secrecy Act. This type of sensitive data can only be made available to researchers after a legal review if meeting the criteria to access this type of sensitive and confidential data. Readers may contact Professor Kristina Alexanderson (kristina.alexanderson@ki.se) for further information regarding the data.

## Abbreviations

ATC: Anatomical therapeutic chemical classification system
CI: Confidence interval
DRG: Diagnosis-related group
EUR: Euros
ICD: International classification of diseases
IQR: Interquartile range
LISA: Longitudinal integration database for health insurance and labour market studies
MiDAS: Micro data for the analysis of social insurance
MS: Multiple sclerosis
NPR: National patient register
PwMS: People with multiple sclerosis
SD: Standard deviation
SEK: Swedish krona
SMSreg: Swedish multiple sclerosis registry
SPDR: Swedish prescribed drug register
VAL: Region Stockholm’s healthcare database

## Code availability

Data management and statistical analyses were performed using SAS v.9.4, except for data management for the cost outcomes in STATA v15.

## References

[1] Giovannoni G, Butzkueven H, Dhib-Jalbut S (2016). Brain health: time matters in multiple sclerosis. Mult. Scler. Relat. Disord..

[2] Jo C (2014). Cost-of-illness studies: concepts, scopes, and methods. Clin. Mol. Hepatol..

[3] Ahlgren C, Oden A, Lycke J (2011). High nationwide prevalence of multiple sclerosis in Sweden. Mult. Scler..

[4] Walton C, King R, Rechtman L (2020). Rising prevalence of multiple sclerosis worldwide: insights from the Atlas of MS, third edition. Mult. Scler..

[5] Rice DP (2000). Cost of illness studies: what is good about them?. Inj. Prev..

[6] Gyllensten H, Wiberg M, Alexanderson K, Friberg E, Hillert J, Tinghog P (2018). Comparing costs of illness of multiple sclerosis in three different years: a population-based study. Mult. Scler..

[7] Gyllensten H, Kavaliunas A, Alexanderson K, Hillert J, Tinghog P, Friberg E (2018). Costs and quality of life by disability among people with multiple sclerosis: a register-based study in Sweden. Mult. Scler. J. Exp. Transl. Clin..

[8] Karampampa K, Gyllensten H, Yang F (2020). Healthcare, sickness absence, and disability pension cost trajectories in the first 5 years after diagnosis with multiple sclerosis: a prospective register-based cohort study in Sweden. Pharmacoecon. Open.

[9] Murley C, Tinghog P, Alexanderson K, Hillert J, Friberg E, Karampampa K (2021). Cost-of-illness progression before and after diagnosis of multiple sclerosis: a nationwide register-based cohort study in Sweden of people newly diagnosed with multiple sclerosis and a population-based matched reference group. Pharmacoeconomics.

[10] Johansson S, Ytterberg C, Gottberg K, Widen Holmqvist L, von Koch L (2009). Use of health services in people with multiple sclerosis with and without fatigue. Mult. Scler..

[11] Gottberg K, Einarsson U, Ytterberg C, Fredrikson S, von Koch L, Holmqvist LW (2008). Use of health care services and satisfaction with care in people with multiple sclerosis in Stockholm County: a population-based study. Mult. Scler..

[12] Ytterberg C, Lundqvist S, Johansson S (2013). Use of health services in people with multiple sclerosis with and without depressive symptoms: a 2-year prospective study. BMC Health Serv. Res..

[13] Ytterberg C, Johansson S, Gottberg K, Holmqvist LW, von Koch L (2008). Perceived needs and satisfaction with care in people with multiple sclerosis: a 2-year prospective study. BMC Neurol..

[14] Chruzander C, Johansson S, Gottberg K (2015). A 10-year population-based study of people with multiple sclerosis in Stockholm, Sweden: use of and satisfaction with care and the value of different factors in predicting use of care. BMC Health Serv. Res..

[15] National Board of Health and Welfare [Socialstyrelsen]. National guidelines care for multiple sclerosis and parkinson's disease: support for steering and management [In Swedish: Nationella riktlinjer Vård vid multipel skleros och Parkinsons sjukdom: Stöd för styrning och ledning] Falun, Sweden 2016. Report No.: 2016:12:1

[16] Papathanasiou A, Saunders L, Sare G (2021). Symptom management of patients with multiple sclerosis in primary care: focus on overlooked symptoms. Br. J. Gen. Pract..

[17] Eriksson I, Komen J, Piehl F, Malmstrom RE, Wettermark B, von Euler M (2018). The changing multiple sclerosis treatment landscape: impact of new drugs and treatment recommendations. Eur. J. Clin. Pharmacol..

[18] Kalincik T, Diouf I, Sharmin S (2021). Effect of disease-modifying therapy on disability in relapsing-remitting multiple sclerosis over 15 years. Neurology.

[19] Thompson AJ, Banwell BL, Barkhof F (2018). Diagnosis of multiple sclerosis: 2017 revisions of the McDonald criteria. Lancet Neurol..

[20] Asche CV, Singer ME, Jhaveri M, Chung H, Miller A (2010). All-cause health care utilization and costs associated with newly diagnosed multiple sclerosis in the United States. J. Manag. Care Pharm..

[21] Onukwugha E, McRae J, Kravetz A, Varga S, Khairnar R, Mullins CD (2016). Cost-of-illness studies: an updated review of current methods. Pharmacoeconomics.

[22] McKay KA, Hillert J, Manouchehrinia A (2019). Long-term disability progression of pediatric-onset multiple sclerosis. Neurology.

[23] Marrie RA, Yu N, Wei Y, Elliott L, Blanchard J (2013). High rates of physician services utilization at least 5 years before multiple sclerosis diagnosis. Mult. Scler..

[24] McKay KA, Friberg E, Razaz N, Alexanderson K, Hillert J (2021). Long-term socioeconomic outcomes associated with pediatric-onset multiple sclerosis. JAMA Neurol..

[25] Wijnands JMA, Kingwell E, Zhu F (2017). Health-care use before a first demyelinating event suggestive of a multiple sclerosis prodrome: a matched cohort study. Lancet Neurol..

[26] Eriksson I, Cars T, Piehl F, Malmstrom RE, Wettermark B, von Euler M (2018). Persistence with dimethyl fumarate in relapsing-remitting multiple sclerosis: a population-based cohort study. Eur. J. Clin. Pharmacol..

[27] Rosenbaum PR, Rubin DB (1983). The central role of the propensity score in observational studies for causal effects. Biometrika.

[28] Karim ME, Pellegrini F, Platt RW, Simoneau G, Rouette J, de Moor C (2020). The use and quality of reporting of propensity score methods in multiple sclerosis literature: a review. Mult. Scler..

[29] Stuart EA (2010). Matching methods for causal inference: a review and a look forward. Stat. Sci..

[30] Austin PC (2011). An introduction to propensity score methods for reducing the effects of confounding in observational studies. Multivar. Behav. Res..

[31] Tarricone R (2006). Cost-of-illness analysis. What room in health economics?. Health Policy.

[32] Drummond MF, Sculpher MJ, Torrance GW, O'Brien BJ, Stoddart GL (2005). Methods for the Economic Evaluation of Health Care Programmes.

[33] Ringborg A, Martinell M, Stalhammar J, Yin DD, Lindgren P (2008). Resource use and costs of type 2 diabetes in Sweden—estimates from population-based register data. Int. J. Clin. Pract..

[34] Eurostat. ECU/EUR exchange rates versus national currencies. https://ec.europa.eu/eurostat/databrowser/view/tec00033/default/table?lang=en (2021). Accessed 05/08/2021

[35] Swedish Association of Local Authorities and Regions [Sveriges Kommuner och Regioner]. Patient fees in outpatient healthcare 2020 [In Swedish: Patientavgifter i öppen hälso-och sjukvård år 2020] (2020). 08-01-2020

[36] Swedish Association of Local Authorities and Regions [Sveriges Kommuner och Regioner]. Patient fees in inpatient healthcare 2020 [In Swedish: Avgifter i sluten vård år 2020] (2020). 08-01-2020

[37] Swedish Association of Local Authorities and Regions [Sveriges Kommuner och Regioner]. Cost per patient Somatic: Retrospective DRG-weights CC-grouped in+outpatient 2012–2019 [Kostnad per patient KPP Somatik: Retrospectiva DRG-vikter CC-grupperat SV+ÖV 2012–2019] [In Swedish]. Swedish Association of Local Authorities and Regions [Sveriges Kommuner och Landsting]. https://skr.se/ekonomijuridikstatistik/statistik/kostnadperpatientkpp/kppsomatik.1077.html (2020). Accessed 05-08-2021

[38] Špacírová Z, Epstein D, Espín J (2022). Are costs derived from diagnosis-related groups suitable for use in economic evaluations? A comparison across nine European countries in the European Healthcare and Social Cost Database. Eur. J. Health Econ..

[39] National Board of Health and Welfare [Socialstyrelsen]. Weight lists for NordDRG [In Swedish: Viktlistor för NordDRG]. https://www.socialstyrelsen.se/utveckla-verksamhet/e-halsa/klassificering-och-koder/drg/viktlistor/ (2020). Accessed 10-08-2020

[40] Swedish Association of Local Authorities and Regions [Sveriges Kommuner och Regioner]. Statistics of healthcare and regional development 2019: Operations and economy in the Regions [In Swedish: Statistik om hälso- och sjukvård samt regional utveckling 2019 Verksamhet och ekonomi i regioner] Stockholm, Statistics section Doeas; 2020 August 2020

[41] Wallstrom S, Ekman I, Omerovic E, Ulin K, Gyllensten H (2019). Cohort study of healthcare use, costs and diagnoses from onset to 6 months after discharge for takotsubo syndrome in Sweden. BMJ Open.

[42] Sabale U, Bodegard J, Sundstrom J (2015). Healthcare utilization and costs following newly diagnosed type-2 diabetes in Sweden: a follow-up of 38,956 patients in a clinical practice setting. Prim. Care Diabetes.

[43] The Dental and Pharmaceutical Benefits Agency [Tandvårds- och läkemedelsförmånsverket (TLV)]. High cost protection [In Swedish: Högkostnadsskyddet]. https://www.tlv.se/lakemedel/hogkostnadsskyddet.html (2021). Accessed 11-02-2022

[44] The Dental and Pharmaceutical Benefits Agency [Tandvårds- och läkemedelsförmånsverket (TLV)]. The Dental and Pharmaceutical Benefits Agency's general guidelines [In Swedish: Tandvårds- och läkemedelsförmånsverkets allmänna råd]. https://www.tlv.se/download/18.467926b615d084471ac3230c/1510316374332/TLVAR_2017_1.pdf. (2017). Accessed 11-02-2022

[45] Statistics Sweden [Statistiska centralbyrån]. Average monthly salary by sector 1992–2020. https://www.scb.se/en/finding-statistics/statistics-by-subject-area/labour-market/wages-salaries-and-labour-costs/salary-structures-whole-economy/pong/tables-and-graphs/average-monthly-salary-by-sector/ (2021). Accessed 05-08-2021

[46] Swedish Tax Authority [Skatteverket]. Total and percentages- income year 2020 [Belopp och procent – inkomstår 2020] [In Swedish]. https://www.skatteverket.se/privat/skatter/beloppochprocent/2020.4.7eada0316ed67d728238ec.html. Accessed 24-11-2021

[47] Swedish MS Association [Svenska MS-Sällskapet (SMSS)]. SMSS Information on Rituximab 210118 [In Swedish: SMSS information om Rituximab 210118]. https://www.mssallskapet.se/wp-content/uploads/2021/03/SMSS-info-om-Rituximab-210118.pdf (2021). Accessed 23-07-2021

[48] FASS.se. Mabthera (Rituximab) [In Swedish]. FASS. https://www.fass.se/LIF/product?userType=2&nplId=19980602000029&docType=30&scrollPosition=600s2019 (2021). Accessed 23-07-2021

[49] Salzer J, Svenningsson R, Alping P (2016). Rituximab in multiple sclerosis: a retrospective observational study on safety and efficacy. Neurology.

[50] FASS.se. Tysabri (Natalizumab) [In Swedish]. FASS. https://www.fass.se/LIF/product?nplId=20040916001115&userType=0&docType=6&scrollPosition=751.33331298828122019#dose (2020). Accessed 23-07-2021

[51] The Dental and Pharmaceutical Benefits Agency [Tandvårds- och läkemedelsförmånsverket (TLV)]. Lemtrada (alemtuzumab) Health economic assessment [Lemtrada (alemtuzumab) Hälsoekonomiskt kunskapsunderlag]. https://www.tlv.se/download/18.467926b615d084471ac339b6/1510316400569/halsoekonomiskt-kunskapsunderlag-lemtrada.pdf (2014). Accessed 23-07-2021

[52] FASS.se. Lemtrada (Alemtuzumab) [In Swedish]. https://www.fass.se/LIF/product?userType=0&nplId=20121223000012&docType=7&scrollPosition=228 (2021). Accessed 23-07-2021

[53] Swedish MS Association [Svenska MS-Sällskapet (SMSS)]. Alemtuzumab [In Swedish]. https://www.mssallskapet.se/wp-content/uploads/2020/03/Alemtuzumab-200207-1.pdf (2020). Accessed 23-07-2021

[54] The Dental and Pharmaceutical Benefits Agency [Tandvårds- och läkemedelsförmånsverket (TLV)]. Arzerra included in the high cost protection with limitations 3266/2014[Arzerra ingår i högkostnadsskyddet med begränsning 3266/2014]. 2015. https://www.tlv.se/beslut/beslut-lakemedel/begransad-subvention/arkiv/2015-01-30-arzerra-ingar-i-hogkostnadsskyddet-med-begransning.html (2021). Accessed 05-08-2021

[55] European Medicines Agency (EMA). Arzerra (ofatumumab). https://www.ema.europa.eu/en/medicines/human/EPAR/arzerra (2021). Accessed 05-08-2021

[56] The Dental and Pharmaceutical Benefits Agency [Tandvårds- och läkemedelsförmånsverket (TLV)]. Assessment for decision-making by county councils: Ocrevus (Ocrelizumab) [Underlag för beslut i landstingen: Ocrevus (okrelizumab)] Dnr 335/2016 (2016). 03-09-2018

[57] FASS.se. Ocrevus (Ocrelizumab) [In Swedish]. https://www.fass.se/LIF/product?userType=0&nplId=20160427000077 (2021). Accessed 23-07-2021

[58] Swedish MS Association [Svenska MS-Sällskapet (SMSS)]. Ocrelizumab information. https://www.mssallskapet.se/wp-content/uploads/2019/12/Ocrelizumab_information.pdf (2017). Accessed 23-07-2021

[59] Eurostat. HICP (2015 = 100) - annual data (average index and rate of change). https://appsso.eurostat.ec.europa.eu/nui/show.do?dataset=prc_hicp_aind&lang=en (2021). Accessed 05-08-2021

[60] Magyari M, Sorensen PS (2020). Comorbidity in multiple sclerosis. Front. Neurol..

[61] Salter A, Lancia S, Cutter G (2021). A propensity-matched comparison of long-term disability worsening in patients with multiple sclerosis treated with dimethyl fumarate or fingolimod. Ther. Adv. Neurol. Disord..

[62] Nguyen TL, Collins GS, Spence J (2017). Double-adjustment in propensity score matching analysis: choosing a threshold for considering residual imbalance. BMC Med. Res. Methodol..

[63] Mihaylova B, Briggs A, O'Hagan A, Thompson SG (2011). Review of statistical methods for analysing healthcare resources and costs. Health Econ..

[64] Andersson E, Persson S, Hallen N (2020). Costs of diabetes complications: hospital-based care and absence from work for 392,200 people with type 2 diabetes and matched control participants in Sweden. Diabetologia.

[65] DiCiccio TJ, Efron B (1996). Bootstrap confidence intervals. Stat. Sci..

[66] Schriefer D, Haase R, Ness NH, Ziemssen T (2021). Cost of illness in multiple sclerosis by disease characteristics—a review of reviews. Expert Rev. Pharmacoecon. Outcomes Res..

[67] Curkendall SM, Wang C, Johnson BH (2011). Potential health care cost savings associated with early treatment of multiple sclerosis using disease-modifying therapy. Clin. Ther..

[68] Tinelli M, Pugliatti M, Antonovici A (2021). Averting multiple sclerosis long-term societal and healthcare costs: the Value of Treatment (VoT) project. Mult. Scler. Relat. Disord..

[69] Ludvigsson JF, Almqvist C, Bonamy AK (2016). Registers of the Swedish total population and their use in medical research. Eur. J. Epidemiol..

[70] Alping P, Piehl F, Langer-Gould A, Frisell T, Group C-MS (2019). Validation of the Swedish Multiple Sclerosis Register: further improving a resource for pharmacoepidemiologic evaluations. Epidemiology.

[71] Ludvigsson JF, Andersson E, Ekbom A (2011). External review and validation of the Swedish national inpatient register. BMC Public Health.

[72] Ludvigsson JF, Svedberg P, Olen O, Bruze G, Neovius M (2019). The longitudinal integrated database for health insurance and labour market studies (LISA) and its use in medical research. Eur. J. Epidemiol..

[73] Brundin L, Kobelt G, Berg J, Capsa D, Eriksson J, European Multiple Sclerosis P (2017). New insights into the burden and costs of multiple sclerosis in Europe: results for Sweden. Mult. Scler..

[74] Smith KA, Burkill S, Hiyoshi A (2021). Comorbid disease burden among MS patients 1968–2012: a Swedish register-based cohort study. Mult. Scler..

[75] Ssegonja R, Alaie I, Philipson A (2019). Depressive disorders in adolescence, recurrence in early adulthood, and healthcare usage in mid-adulthood: a longitudinal cost-of-illness study. J. Affect. Disord..

[76] Swedish Neuro Registry [Svenska Neuroregister]. Annual Report 2019 [In Swedish: Årsrapport 2019]. https://www.neuroreg.se/media/ulql2mip/multipel-skleros-%C3%A5rsrapport-2019.pdf (2020). Accessed 27-01-2021

[77] Organisation for Economic Cooperation and Development (OECD) and the European Union (2018). Health at a Glance: Europe 2018: 8: State of Health in the EU Cycle.

[78] Organisation for Economic Cooperation and Development (OECD) (2013). Consultations with Doctors.

[79] Johansson N, Jakobsson N, Svensson M (2018). Regional variation in health care utilization in Sweden—the importance of demand-side factors. BMC Health Serv. Res..

[80] Martinelli V, Rodegher M, Moiola L, Comi G (2004). Late onset multiple sclerosis: clinical characteristics, prognostic factors and differential diagnosis. Neurol. Sci..

[81] Buscarinu MC, Reniè R, Morena E (2022). Late-onset MS: disease course and safety-efficacy of DMTS. Front. Neurol..

[82] Murley C, Friberg E, Hillert J, Alexanderson K, Yang F (2019). Validation of multiple sclerosis diagnoses in the Swedish National Patient Register. Eur. J. Epidemiol..

