# Supplementary material for: Excess costs of multiple sclerosis: a register-based study in Sweden
Source: Eur J Health Econ. 2022 Nov 23;24(8):1357–71. doi: 10.1007/s10198-022-01547-6 (PMC9685028; doi:10.1007/s10198-022-01547-6)
Supplement: Supplementary file 1 — Supplementary file1 (PDF 350 kb) [file 10198_2022_1547_MOESM1_ESM.pdf]

Excess costs of multiple sclerosis: A register-based study in Sweden

Electronic Supplementary Material

Online Resource 1: Information regarding the register data obtained and linked

Authority responsible for the register	Register
**Region Stockholm**	• **Swedish MS Registry** (SMSReg)[1,2]<br><br>The SMSReg is a voluntary nationwide MS-specific clinical register used by neurologists, established in 2001. Approximately 80.1% of the PwMS in Sweden are included in the register.[3] Information extracted regarded the dates for MS diagnosis and estimated onset as well as detailed information on the MS DMTs administered within healthcare which were unavailable in the SPDR.<br><br>• **Region Stockholm's healthcare database** (VAL)<br><br>The VAL is the healthcare database including information on primary healthcare contacts with both public and private providers within Stockholm County that have financing agreements with Region Stockholm. Region Stockholm is responsible for the organisation of healthcare and funding healthcare services for the residents of Stockholm County.[4] Primary healthcare is primarily financed from tax revenues, with modest flat-rate patient fees for healthcare visits (copayments). There is a government-imposed cap on the out-of-pocket copayments within a 12-month period for patient fees for primary and specialised outpatient healthcare, where after reaching the ceiling the patient pays no further fees.[4,5] The data extracted regarded the date of contact, diagnoses, type of contact, and type of healthcare professional.
**Statistics Sweden**	• **Longitudinal Integration Database for Health Insurance and Labour Market Studies** (LISA)[6,7]<br><br>The LISA contains yearly microdata of all individuals (>15 years) resident in Sweden. LISA was used to identify the source population of working-aged individuals registered as living in Stockholm County and information on the sociodemographic characteristics used in the propensity score (sex, age, country of birth, degree of urbanisation of municipality of residence, educational level, and family composition).
**National Board of Health and Welfare**	• **National Patient Register** (NPR)[8,9]<br><br>The NPR contains information on inpatient and specialised outpatient healthcare visits throughout Sweden. The register has had nationwide coverage of all inpatient admissions since 1987 and of specialised outpatient visits with public and private providers since 2001. Specialised outpatient and inpatient healthcare in Sweden are predominately financed by tax revenues. Specialised outpatient and inpatient healthcare have separate flat-rate patient fee schedules, with a government-imposed maximum cap on the copayments for patient fees for specialised outpatient as well as primary healthcare.[4,5,10] The information for each healthcare visit extracted included the date, diagnoses, and the diagnosis-related group (DRG) code.<br><br>• **Swedish Prescribed Drug Register** (SPDR)[11]<br><br>The SPDR provided nationwide information of the dates, substances, and costs for all prescribed drugs dispensed at community pharmacies in Sweden since 2005. A nationwide high cost protection covers most prescribed drugs by progressively discounting the patient copayments with increasing out-of-pocket expenditure, up to a maximum copayment within a 12-month period.[4,12]<br><br>• **Cause of Death Register**[13,14]

	The register contains information on the date of death.
**Swedish Social Insurance Agency**	• **Micro Data for the Analysis of Social Insurance register** (MiDAS)[15]<br><br>The MiDAS contains detailed information on the universal social insurances sickness absence (SA) and disability pension (DP).[15] Contributions to these insurances are mandatory and the benefits are primarily financed through employer payroll fees (social security contribution).[4] All residents in Sweden with work-related income are eligible for SA when having temporarily reduced work capacity due to disease or injury. Net days from SA spells >14 days were included. DP can be granted for long-term or permanent reductions of work capacity due to disease of injury. Both SA and DP can be for full-time (100%) or part-time (25, 50, or 75%), with simultaneous benefits possible up to 100% of ordinary working hours. Accordingly, information on the net days of SA and DP were obtained to account for this possibility.

Abbreviations: DP: Disability pension; DRG: Diagnosis-related group; LISA: Longitudinal Integration Database for Health Insurance and Labour Market Studies; MiDAS: Micro Data for the Analysis of Social Insurance register; MS: Multiple sclerosis; NPR: National Patient Register; SA: Sickness absence SMSReg: Swedish Multiple Sclerosis Registry; SPDR: Swedish Prescribed Drug Register; VAL: Region Stockholm's healthcare database.

Online Resource 2: Costing matrix for primary healthcare with a base cost of a visit with a physician at a primary healthcare clinic

Healthcare professional	Type of healthcare contact	Unit cost (SEK)[116]	Unit cost (EUR)
**Physician** (general medicine)	Visit at a healthcare clinic	**1879 SEK (base cost)**	**179 EUR**
	Home visit	2x base cost (3758 SEK)	358 EUR
	Distance contact	1/3 base cost (626 SEK)	58 EUR
**Nurse or other healthcare professional**[2]	Visit at a healthcare clinic	40% base cost (752 SEK)	72 EUR
	Home visit	2x 40% base cost (1503 SEK)	143 EUR
	Distance contact	1/3 of 40% base cost (251 SEK)	24 EUR

[1] All unit prices from 2019 are inflated to 2020 Swedish prices using annual Harmonised Indices of Consumer Prices (HICP) for healthcare available from Eurostat.[17] Conversion from Swedish Krona (SEK) to Euros (EUR) was performed via the 2020 annual exchange rate of 10.4848 when applying this matrix.[18]

[2] Excluding physicians

Abbreviations: EUR: Euros; HICP: Harmonised Indices of Consumer Prices; SEK: Swedish Krona.

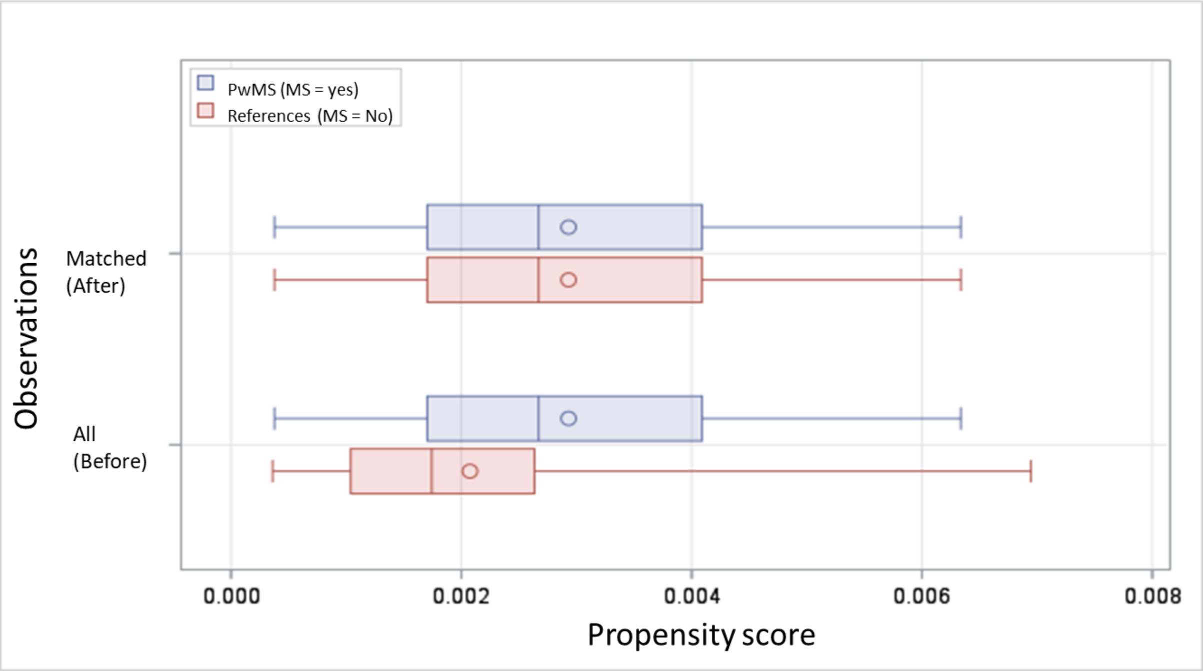

Online Resource 3: Box plots of the propensity score distribution of the PwMS (blue) and references without MS (red) comparing distributions of the propensity score of all included in the potential reference pool before matching (All), and those references included in the matched reference group after matching on the propensity score 1:10 (Matched).

Abbreviations: MS: Multiple sclerosis; PwMS: People with multiple sclerosis

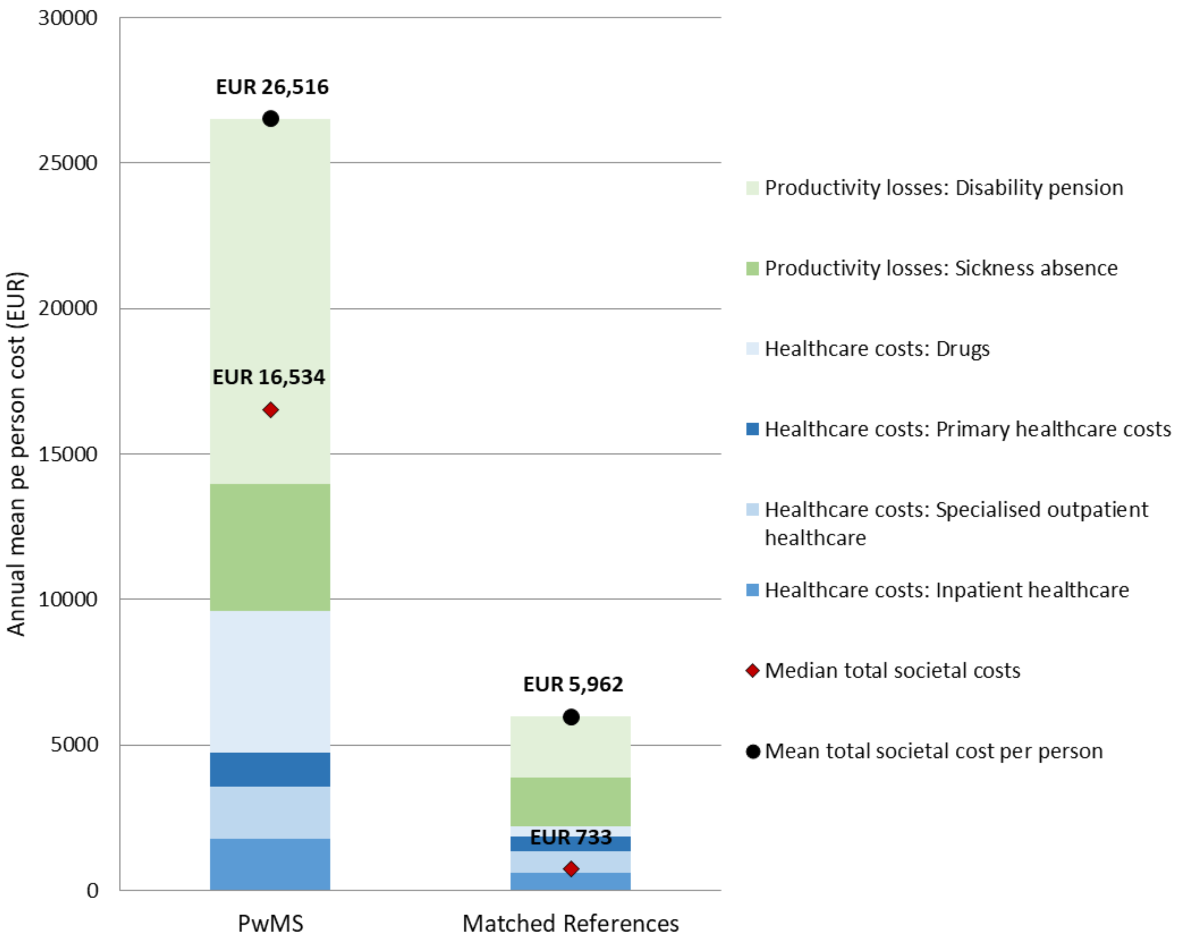

Online Resource 4: Annual per person mean societal costs among people with multiple sclerosis and propensity score matched references without multiple sclerosis.

Notes: Blue refers to cost components of the healthcare costs and green for the productivity loss components. The annual exchange rate for 2020 from SEK to EUR from Eurostat was 10.4848.[18] Prior to currency conversion, if required, the unit costs were inflated to 2020 Swedish prices using the annual HICP for healthcare available from Eurostat.[17]

Abbreviations: EUR: Euros HICP: Harmonised indices of consumer prices; PwMS: People with multiple sclerosis; SEK: Swedish Krona.

Online Resource 5: Mean and median per person costs[a] in Euros for all of the PwMS and the population-based propensity-score matched references without MS, respectively, as well as the mean differences (excess costs of MS) with bootstrapped 95% CIs between the PwMS and matched references.

	PwMS (n=2806)							References (n=28,060)							Mean difference		
**Cost category**	**Users (n)**	**Users (%)**	**Mean**	**SD**	**(95% CI)**	**Median**	**(IQR)**	**Users (n)**	**Users (%)**	**Mean**	**SD**	**(95% CI)**	**Median**	**(IQR)**	**Mean difference**	**(95% CI)**	**p- value**
**Total societal costs**	2758	98.29	26,516	26,729	(25,527 to 27,506)	16,534	(3859 to 48,841)	23,086	82.27	5962	14,782	(5789 to 6135)	733	(156 to 2875)	20,554	(19,552 to 21,549)	<0.0001
**Healthcare costs**	2748	97.93	9595	10,955	(9189 to 10,000)	5754	(2561 to 13,844)	23,002	81.97	2214	5821	(2146 to 2282)	671	(129 to 2039)	7381	(6991 to 7816)	<0.0001
Patient copayments	2698	96.15	304	260	(294 to 314)	283	(157 to 401)	23000	81.97	213	1158	(199 to 226)	119	(29 to 253)	91	(73 to 107)	<0.0001
Publicly financed	2733	97.40	9291	10851	(8889 to 9692)	5405	(2376 to 13,550)	21436	76.39	2001	5577	(1936 to 2067)	538	(72 to 1754)	7289	(6906 to 7720)	<0.0001
Inpatient healthcare	434	15.47	1780	6266	(1548 to 2012)	0	(0 to 0)	2068	7.37	621	3803	(577 to 666)	0	(0 to 0)	1159	(928 to 1394)	<0.0001
Specialised outpatient healthcare	2234	79.62	1785	2272	(1701 to 1869)	1067	(358 to 2374)	13,725	48.91	737	1615	(718 to 756)	0	(0 to 828)	1048	(959 to 1133)	<0.0001
Primary healthcare	2130	75.91	1181	3266	(1060 to 1302)	397	(81 to 1101)	18,381	65.51	486	965	(475 to 498)	198	(0 to 640)	695	(585 to 832)	<0.0001
Total drugs	2685	95.69	4848	7240	(4580 to 5116)	1728	(820 to 5806)	20,030	71.38	370	2506	(340 to 399)	43	(0 to 157)	4479	(4224 to 4769)	<0.0001
DMTs[b]	1913	68.18	4262	6371	(4026 to 4497)	1541	(0 to 5381)	n.a.									
Non-DMT drugs	2473	88.13	587	3521	(456 to 717)	160	(38 to 536)	20,030	71.38	370	2506	(340 to 399)	43	(0 to 157)	217	(107 to 390)	0.0015
**Productivity losses**	1371	48.86	16,922	22,166	(16,101 to 17,742)	0	(0 to 35,852)	4316	15.38	3748	11,966	(3608 to 3888)	0	(0 to 0)	13,173	(12,325 to 14,019)	<0.0001
**SA**	649	23.13	4385	11,706	(3952 to 4819)	0	(0 to 0)	3123	11.13	1658	6908	(1577 to 1739)	0	(0 to 0)	2727	(2278 to 3186)	<0.0001
**DP**	878	31.29	12,536	20,863	(11,764 to 13,308)	0	(0 to 27,149)	1308	4.66	2090	9985	(1973 to 2207)	0	(0 to 0)	10,446	(9636 to 11,206)	<0.0001

[a] The mean and median costs were calculated for all PwMS and references respectively, not just resource users. The annual exchange rate for 2020 from SEK to EUR from Eurostat was 10.4848.[18] Prior to currency conversion, if required, the unit costs were inflated to 2020 Swedish prices using the annual HICP for healthcare available from Eurostat.[17]

[b] DMTs sourced from either the SPDR (ATC codes: L03AB07; L03AB08; L03AB13; L03AX13; L04AA27; L04AA31; L04AA40; L04AC01; L04AX07; and N07XX09) or the SMSReg (ATC codes: L04AA23; L01XC02; L01XC10; L04AA34; L04AA36; and L01DB07). No L01DB07 was found in the SMSReg for the study cohort in 2018.

Abbreviations: ATC: Anatomical Therapeutic Chemical Classification System; CI: Confidence intervals; DMTs: Disease modifying therapies; EUR: Euros; DMTs: Disease modifying therapies; DP: Disability Pension; IQR: Interquartile range; MS: Multiple sclerosis PwMS: People with multiple sclerosis; SA: Sickness absence; SD: Standard deviation; SMSReg: Swedish Multiple Sclerosis Registry; SPDR: Swedish Prescribed Drug Register

Online Resource 6: Numbers and proportions of users of resources among the PwMS and the propensity score matched references without MS

	PwMS (n=2806)		References (n=28,060)		P-value [a]
	Users (n)	Users (%)	Users (n)	Users (%)	
**Total societal costs**	2758	98.29	23,086	82.27	<0.0001
**Healthcare**	2748	97.93	23,002	81.97	<0.0001
Patient out-of-pocket copayments for healthcare visits and drugs	2698	96.15	23,000	81.97	<0.0001
Publicly financed costs for healthcare visits and drugs	2733	97.40	21,436	76.39	<0.0001
**Inpatient healthcare**	434	15.47	2068	7.37	<0.0001
**Specialised outpatient healthcare**	2234	79.62	13,725	48.91	<0.0001
**Primary healthcare**	2130	75.91	18,381	65.51	<0.0001
**Type of visit**					
Clinic (incl. cancelled)	2085	74.31	18,313	65.26	<0.0001
Home/offsite	388	13.83	325	1.16	<0.0001
Distance	106	3.78	1049	3.74	0.9169
**Type of professional**					
Physician	1865	66.46	16,415	58.50	<0.0001
Nurse	713	25.41	4465	15.91	<0.0001
Other healthcare professionals	1173	41.80	7579	27.01	<0.0001
**Type of professional (detailed)**					
Physician	1865	66.46	16,415	58.50	<0.0001
Nurse	713	25.41	4465	15.91	<0.0001
Physiotherapist	580	20.67	2760	9.84	<0.0001
Occupational therapist	376	13.40	515	1.84	<0.0001
Nurse assistant	166	5.92	608	2.17	<0.0001
Other healthcare professionals	747	26.62	5395	19.23	<0.0001
**Drugs**	2685	95.69	20,030	71.38	<0.0001
DMTs [b]	1913	68.18		n.a.	
Non-DMT drugs	2473	88.13	20,030	71.38	<0.0001
**Productivity losses**	1371	48.86	4316	15.38	<0.0001
**Sickness absence**	649	23.13	3123	11.13	<0.0001
**Disability pension**	878	31.29	1308	4.66	<0.0001

[a] P-value calculated with chi-square tests.

[b] DMTs identified from either the SMSReg (ATC codes: L04AA23; L01XC02; L01XC10; L04AA34; L04AA36; L01DB07) or SPDR (ATC codes: L03AB07; L03AB08; L03AB13; L03AX13; L04AA27; L04AA31; L04AA40; L04AC01; L04AX07; and N07XX09).

Abbreviations: ATC: Anatomical Therapeutic Chemical Classification System; DMTs: Disease modifying therapies; MS: Multiple sclerosis; PwMS: People with multiple sclerosis; SMSReg: Swedish Multiple Sclerosis Registry; SPDR: Swedish Prescribed Drug Register

Online Resource 7: Number and percentage of users of primary healthcare, mean per person costs[a] in Euros with 95% CIs among all of the PwMS and the population-based propensity-score matched references without MS, respectively, and the mean differences (excess costs of MS) with bootstrapped 95% CIs between the PwMS and matched references.

	PwMS (n=2806)						References (n=28,060)						Mean difference		
**Cost outcome**	**Users (n)**	**Users (%)**	**Mean**	**(95% CI)**	**Median**	**(IQR)**	**Users (n)**	**Users (%)**	**Mean**	**(95% CI)**	**Median**	**(IQR)**	**Mean difference**	**(95% CI)**	**p- value**
**Region Stockholm's cost[b]**	2130	75.91	1129	(1009 to 1249)	358	(72 to 1004)	18,381	65.51	448	(437 to 459)	179	(0 to 574)	681	(571 to 816)	<0.0001
**Type of healthcare visit**															
Clinic	2085	74.31	662	(625 to 698)	358	(0 to 824)	18,313	65.26	424	(417 to 432)	179	(0 to 538)	237	(197 to 274)	<0.0001
Home/offsite	388	13.83	465	(355 to 575)	0	(0 to 0)	325	1.16	21	(13 to 29)	0	(0 to 0)	444	(342 to 560)	<0.0001
Distance contacts/telephone	106	3.78	2	(2 to 3)	0	(0 to 0)	1049	3.74	2	(2 to 3)	0	(0 to 0)	0	(-1 to 1)	0.8297
**Type of healthcare professional**															
Physician	1865	66.46	376	(356 to 395)	179	(0 to 538)	16,415	58.50	290	(285 to 294)	179	(0 to 358)	86	(66 to 107)	<0.0001
Nurse	713	25.41	230	(176 to 284)	0	(0 to 72)	4465	15.91	32	(28 to 36)	0	(0 to 0)	198	(146 to 251)	<0.0001
Other healthcare professionals	1173	41.80	523	(442 to 604)	0	(0 to 287)	7579	27.01	126	(120 to 133)	0	(0 to 72)	397	(325 to 493)	<0.0001
**Type of healthcare professional (detailed)**															
Physician	1865	66.46	376	(356 to 395)	179	(0 to 538)	16,415	58.50	290	(285 to 294)	179	(0 to 358)	86	(66 to 107)	<0.0001
Nurse	713	25.41	230	(176 to 284)	0	(0 to 72)	4465	15.91	32	(28 to 36)	0	(0 to 0)	198	(146 to 251)	<0.0001
Physiotherapist	580	20.67	186	(164 to 208)	0	(0 to 0)	2760	9.84	50	(47 to 52)	0	(0 to 0)	136	(113 to 159)	<0.0001
Occupational therapist	376	13.40	96	(81 to 110)	0	(0 to 0)	515	1.84	5	(4 to 6)	0	(0 to 0)	90	(77 to 107)	<0.0001
Nurse assistant	166	5.92	132	(64 to 200)	0	(0 to 0)	608	2.17	9	(4 to 14)	0	(0 to 0)	123	(68 to 208)	0.0004
Other healthcare professionals	747	26.62	110	(97 to 122)	0	(0 to 72)	5395	19.23	62	(60 to 65)	0	(0 to 0)	48	(35 to 61)	<0.0001

[a] The first registered healthcare professional per contact is used. Primary healthcare costs are excluding the patient out of pocket copayment in the form of visit fees. Mean costs are for all PwMS and references respectively, not just resource users. The annual exchange rate for 2020 from SEK to EUR from Eurostat was 10.4848.[18] Prior to currency conversion, if required, the unit costs were inflated to 2020 Swedish prices using the annual HICP for healthcare available from Eurostat.[17]

[b] Patient out-of-pocket copayments were unable to be disentangled due to the annual ceiling payments. Accordingly, all costs presented in this table refer to the publicly financed costs for primary healthcare.

Abbreviations: CI: Confidence interval; EUR: Euros; HICP: Harmonised Index of Consumer Prices; IQR: Inner quartile range; MS: Multiple sclerosis; PwMS: People with multiple sclerosis SEK: Swedish Krona

Reference list for the Supplementary Material

1. Hillert J, Stawiarz L. The Swedish MS registry - clinical support tool and scientific resource. *Acta neurologica Scandinavica.* 2015;132(199):11-19.
2. Alping P, Piehl F, Langer-Gould A, Frisell T, Group C-MS. Validation of the Swedish Multiple Sclerosis Register: Further Improving a Resource for Pharmacoepidemiologic Evaluations. *Epidemiology.* 2019;30(2):230-233.
3. Swedish Neuro Registry [Svenska Neuroregister]. Annual Report 2019 [In Swedish: Årsrapport 2019] 2020; https://www.neuroreg.se/media/ulql2mip/multipel-skleros-%C3%A5rsrapport-2019.pdf. Accessed 27-01-2021.
4. European Observatory on Health Systems and Policies. Health Systems in Transition (HiT) profile of Sweden. 2012; https://eurohealthobservatory.who.int/monitors/health-systems-monitor/countries-hspm/hspm/sweden-2012. Accessed 24-11-2021.
5. Swedish Association of Local Authorities and Regions [Sveriges Kommuner och Regioner]. *Patient fees in outpatient healthcare 2020* [In Swedish: Patientavgifter i öppen hälso-och sjukvård år 2020] 08-01-2020 2020.
6. Statistics Sweden [Statistiska centralbyrån]. *Longitudinal integration database for sick leave and labour market studies (LISA) 1990-2013.* [In Swedish: Longitudinell integrationsdatabas för sjukförsäkrings- och arbetsmarknadsstudier (LISA) 1990-2013.]. Örebro, Sweden: Statistics Sweden [Statistika centralbyrån];2016.
7. Ludvigsson JF, Svedberg P, Olen O, Bruze G, Neovius M. The longitudinal integrated database for health insurance and labour market studies (LISA) and its use in medical research. *Eur J Epidemiol.* 2019;34(4):423-437.
8. Ludvigsson JF, Andersson E, Ekbom A, et al. External review and validation of the Swedish national inpatient register. *BMC Public Health.* 2011;11:450.
9. National Board of Health and Welfare [Socialstyrelsen]. The National Patient Register. 2019; https://www.socialstyrelsen.se/en/statistics-and-data/registers/register-information/the-national-patient-register/. Accessed 24-11-2021.
10. Swedish Association of Local Authorities and Regions [Sveriges Kommuner och Regioner]. *Patient fees in inpatient healthcare 2020* [In Swedish: Avgifter i sluten vård år 2020] 08-01-2020 2020.
11. Wettermark B, Hammar N, Fored CM, et al. The new Swedish Prescribed Drug Register--opportunities for pharmacoepidemiological research and experience from the first six months. *Pharmacoepidemiol Drug Saf.* 2007;16(7):726-735.
12. The Dental and Pharmaceutical Benefits Agency [Tandvårds- och läkemedelsförmånsverket (TLV)]. High cost protection [In Swedish: Högkostnadsskyddet]. 2021; https://www.tlv.se/lakemedel/hogkostnadsskyddet.html. Accessed 11-02-2022.
13. National Board of Health and Welfare [Socialstyrelsen]. The Cause of Death Register [In Swedish: Dödsorsaksregistret]. 2019; https://www.socialstyrelsen.se/statistik-och-data/register/alla-register/dodsorsaksregistret/. Accessed 11-02-2022.
14. Brooke HL, Talback M, Hornblad J, et al. The Swedish cause of death register. *Eur J Epidemiol.* 2017;32(9):765-773.
15. Swedish Social Insurance Agency [Försäkringskassan]. *MiDAS: Microdata for the analysis of sickness absence and disability pension* [In Swedish: Sjukpenning och rehabiliteringspenning (MiDAS: Sickness benefit and rehabilitation benefit]. Stockholm Swedish Social Insurance Agency [Försäkringskassan], ;2011.
16. Swedish Association of Local Authorities and Regions [Sveriges Kommuner och Regioner]. *Statistics of healthcare and regional development 2019: Operations and economy in the Regions* [In Swedish: Statistik om hälso- och sjukvård samt regional utveckling 2019 Verksamhet och ekonomi i regioner] Stockholm August 2020 2020.
17. Eurostat. HICP (2015 = 100) - annual data (average index and rate of change). 2021; https://appsso.eurostat.ec.europa.eu/nui/show.do?dataset=prc_hicp_aind&lang=en. Accessed 05-08-2021, 2021.
18. Eurostat. ECU/EUR exchange rates versus national currencies. 2021; https://ec.europa.eu/eurostat/databrowser/view/tec00033/default/table?lang=en. Accessed 05/08/2021, 2021.
